# What drives patients’ acceptance of Digital Therapeutics? Establishing a new framework to measure the interplay between rational and institutional factors

**DOI:** 10.1186/s12913-023-09090-7

**Published:** 2023-02-10

**Authors:** Alessandro Carrera, Francesca Zoccarato, Margherita Mazzeo, Emanuele Lettieri, Giovanni Toletti, Simona Bertoli, Gianluca Castelnuovo, Emanuele Fresa

**Affiliations:** 1 Polytechnic University of Milan, School of Management, Milan, Italy; 2 IRCCS Istituto Auxologico Italiano, Milan, Italy; 3 Department of Food, Environmental and Nutritional Sciences, University of Milan, Milan, Italy; 4 Department of Psychology, Catholic University, Milan, Italy

**Keywords:** Diffusion, Healthcare, Digital therapeutics, Acceptance, Institutional theory

## Abstract

**Background:**

The rising incidence of chronic diseases among the population, further exacerbated by the phenomenon of aging, is a primary concern and a serious challenge for the healthcare systems worldwide. Among the wide realm of health digital technologies, the rise of Digital Therapeutics (DTx), which are medical devices able to deliver evidence-based treatments to manage and treat diseases, opens new opportunities. However, their diffusion and usage are still fragmented among countries. As the diffusion results from the adoption of technology from a social system and individual acceptance, this study aims to design and test a theoretical model that investigates the intention to use DTx, with a particular focus on the treatment of obesity, as a widespread and burdensome chronic condition.

**Methods:**

This research is built on 336 answers coming from a survey to test the proposed model, which consists of a combination of organizational mechanisms, derived from Institutional Theory, and rational factors, derived from the Technology Acceptance Model (TAM). The survey has been delivered to patients and former patients of Istituto Auxologico Italiano, a hospital with several locations in northern Italy, recognized as a center of excellence for the treatment of obesity.

**Results:**

The analyses of the answers, performed through the Structural Equation Modelling (SEM) technique, confirmed the influence of the Perceived Usefulness on Intention To Use, and of the Perceived Ease Of Use on the Perceived Usefulness, confirming the validity of the assumptions derived from the TAM. On the other hand, institutional factors were introduced as antecedents of the Perceived Usefulness, and the Perceived Ease Of Use. Results show that the Regulative Pillar influences both the TAM constructs, the Normative Pillar (peer influence) has a positive effect only on the Perceived Usefulness, and finally, the Cultural Pillar impacts the Perceived Ease Of Use.

**Conclusion:**

This study allows filling the knowledge gap regarding the usage of the Institutional as a means to predict individuals’ intentions. Moreover, managerial contributions are available as the results have been operationalized into practical advice to managers and healthcare professionals to foster the adoption, and thus the diffusion, of Digital Therapeutics.

**Supplementary Information:**

The online version contains supplementary material available at 10.1186/s12913-023-09090-7.

## Background

According to the (World Health Organization), 1 out of 10 people worldwide is obese [1]. Obesity constitutes a global and wicked societal challenge, as it reduces the quality of life by negatively impacting both physical and psychosocial functioning [2], increases the risk for other chronic and non-communicable diseases, and ultimately affects healthcare economic resources. Indeed, it has been estimated that the cost for an obese patient can be 50% higher than the one for a patient with an ideal weight [3].

For these reasons, comprehensive obesity management is an urgent yet complex challenge [4]. Specifically, a multidisciplinary approach integrating a psychological approach to the more traditional physical and nutritional interventions has been suggested by medical guidelines as the most effective option [5], pinpointing cognitive behavioral therapy for changing behaviors to successfully lose and maintain weight. Indeed, it acts through a combination of cognitive and behavioral therapies modifying patients’ cognitive processes and, consequently, behaviors [4].

In this setting, the area of research involving technology for behavioral change is rapidly growing. Past research involves periodic prompts [6], psychologist coaching programs based on cognitive-behavioral therapy (CBT) modules [7], telehealth trials with telephone and text support [8], and smartphone-based physical activity coaching interventions which were able to significantly increase daily physical activity [9]. More recently, Digital Therapeutics (DTx) are gaining momentum thanks to their benefits as they have proven not only to be effective but also cost-efficient [10, 11], thus departing from the results of previous interventions [12]. DTx are evidence-based therapeutic interventions driven by high-quality software to treat a medical disorder or disease (DTA, 2020), and they are in the form of apps, web-based systems, videogames, virtual reality (VR), text messages, social media platforms, and others [13]. Thus, they are also able to guarantee continuity of care for the patients and real-world data for physicians and providers.

Due to their benefits, DTx are raising strong interest as of January 2021, 136 randomized clinical trials of DTx were either ongoing or concluded [14]. Anyway, proper ecosystem building for DTx is facing some challenges.

Firstly, being software without hardware, each Digital Therapeutic is *Software as a Medical Device* (SaMD) according to the classification provided by FDA (FDA, 2018) [15]. Thus, the demonstration of efficacy through clinical trials together with Real World Evidence (RWE) [16]. Additionally, the regulatory framework also deserves attention [17]. For instance, while in Italy proper regulation is still lacking, Germany’s Digital Healthcare Act (Digitale-Versorgung-Gesetz — DVG) [18, 19] and the Pre-cert program in the U.S. represent streamlined and efficient paths dedicated to DTx [20]. Moreover, reimbursement represents a crucial aspect that is not always straightforward, and, also, in this case, some countries are developing their systems to face this challenge, while other countries are still lagging [17].

Besides the abovementioned issues, the know-how for DTx development represents a further aspect that might affect their diffusion: indeed, the know-how for DTx development is lacking among pharmaceutical incumbents, thus requiring diverse and various kinds of cooperation with different actors in the field [21].

Notwithstanding, a more individual perspective should be taken into account when dealing with DTx. Despite these benefits, chronic patients’ acceptance of DTx cannot be taken for granted because of the Copernican revolution of delivering therapies through apps, which poses great empowerment to patients’ choices. Acceptance is a key factor to predict the diffusion over time of health digital technologies [22], which is fundamental to size the positive effects they can have on obese patients’ health. Specifically, this study aims at shedding new light on the key elements driving the acceptance of DTx to fully unlock their potential. Indeed, a lack of acceptability by users of digital technologies could be inefficient in terms of both economic advantage and clinical outcomes [23].

The paper is structured as follows. In the next sub-section (Model antecedents) the literature on addressing the topic of acceptance of new technology is reviewed to highlight the gaps and develop a research framework comprising elements of diverse theories and able to support the subsequent empirical analysis. In the Methods section, the methodology and data collection and analysys are presented. Results section outlines the findings of the empirical investigation and discusses the major achievements of the paper. Finally, the last section drowns the final discussion about the value for researchers and managers of the main results.

### Model antecedents

#### Rational factors

One of the most diffused models is the Technology Acceptance Model (TAM) [24, 25], which is derived from the Theory of Reasoned Actions (TRA) [26]. TAM has its deepest roots in social psychology and aims to analyze how external variables influence an individual’s beliefs, attitudes, and intentions. TAM continues to be one of the most widely used models for defining and predicting user acceptance of technology. According to the model, there are two main predictors of the user’s intention to adopt an innovation: Perceived Usefulness (PU) and Perceived Ease of Use (PEU).

PU is defined as “the degree to which a person believes that using a particular system would enhance his or her job performance” [24, 25]. PU defines the productivity and the effectiveness, that the individual perceived in using the technology for his/her work.

PEU refers to “the degree to which a person believes that using a particular system would be free of effort” [24, 25]. This means that the perception of the person who uses the technology is free from physical and mental pain.

According to TAM, PEU and PU are affected by external variables such as design and user features, task characteristics, nature of development and implementation process, political influences, and others. The two variables are considered the main predictors of usage intention and, consequently, of usage behavior.

As mentioned above, TAM is one of the most exploited approaches to investigate the dynamics of acceptance of information system technologies in healthcare [27], together with its extended versions TAM2 [28] and TAM3 [29], which include additional antecedents such as social influence, namely Subjective Norm, and cognitive tools. More specifically, it has been used to analyze both patients’ and physicians’ acceptance of digital technologies in healthcare, such as telemedicine [30] and Electronic Medical Records (EMR) [31].

#### Institutional factors

An alternative perspective sees actions as a result of irrationalities coming from the institutional environment and not as based upon a rational process. The establishment, evolution, and decline over time of institutional structures as guidelines for actions are explored in the Institutional Theory [32]. In this perspective, organizations are built over a set of values, norms, and beliefs which affect and constrain actions over time. In this way, institutions constrain the options available to individuals and collectives. From Scott’s definition, institutions are built upon three main pillars: regulative (coercive), normative, and cultural-cognitive.

- The Regulative Pillar (RP) is based on the coercion institutions can exert to constrain and regulate the actors’ behavior. This exists as there are rules, norms, and regulations that establish what can be done and sanctions for breaches when rules are not respected.
- The Normative Pillar (NP) exploits the expectations and norms elaborated by social groups about what could be appropriate behavior in some circumstances, i.e., in the organization. Organizations can exert normative influence through forms of peer influence, which is meant to align individuals to the belief of the necessity of the new technology.
- The Cultural-cognitive Pillar (CP) includes the common mental schemes and the symbolic representations shared among the social group. The more the idea that the status quo must be changed, the more the individual is likely to adhere to the cultural change.

For what concerns the healthcare domain, researchers have previously studied the effects of institutional pressures on electronic health records (EHR) adoption in the hospitalized setting, proposing a framework highlighting key constructs such as Cause, Constituents, Content, Context, and Control [33].

Most of the studies focus on the physicians’ perspective highlighting how they perceive the institutional forces and how they behave in response to such pressures [34]. However, the point of view of patients has been largely overlooked. In particular, chronic patients who need a life-lasting care path with the involvement of a high number of different professionals might experience strong institutional pressure from the health institution (e.g., the hospital).

## Methods

In this section, the model used to study the diffusion of DTx is presented with its theoretical underpinnings. Past studies mainly focus on the adoption of technology from a social system rather than its diffusion and can be divided between those which explore the adoption of the technology through a general classification of enablers and barriers [35–40] and those which apply validated models in the literature [33, 41–45]. In the latter case, the most frequently used models are built over very different, if not even contrasting, hypotheses. Indeed, on the one hand, theories like the Technology Acceptance Model [24, 25] identify rational factors as the driving force of individual behavior. On the other hand, the Institutional Theory [32] suggests that individual behavior is limited by the set of institutional rules, expectations, culture, and meaning systems.

The proposed model explores the institutional effects on the rational behavior of patients. The hospital setting has been a testing place both for the acceptance model and for institutional explanations, although no evidence has been collected on the results of institutional pressure on patients. Therefore, this study will be able to test the integrative explanatory power of the two theories.

### Model design

In recent years, the institutional theory included the importance of a degree of rational behavior while TAM included the importance of the social norm, yet there is not a strong integration of the two theories in the past literature. Hence, the research model that has been developed aims to investigate the interplay between the organizational and individual mechanisms, represented by the constructs in Table 1, which could influence the continued use of digital therapeutics among obese patients.

**Table 1:** Theoretical sources for selected constructs

Construct	Theory	Theory Conceptualization
Intention to Use	Technology Acceptance Model	Davis [24]
Perceived Usefulness	Technology Acceptance Model	Davis [24]
Perceived Ease of Use	Technology Acceptance Model	Davis [24]
Regulative Pillar	Institutional Theory	Scott [32]
Normative Pillar	Institutional Theory	Scott [32]
Cultural Cognitive Pillar	Institutional Theory	Scott [32]

The selected constructs come from both Davis’ [25] TAM and Institutional Theory. The former are the three basic and fundamental elements of TAM. For what concerns the institutional factors, Scott [32] conceived institutions as made of pillars limiting the rational assessment and directing actors’ behavior. These are regulative, normative, and cultural pillars, which in turn can be exploited by the organizations to exert the following influences.

**Fig. 1:**
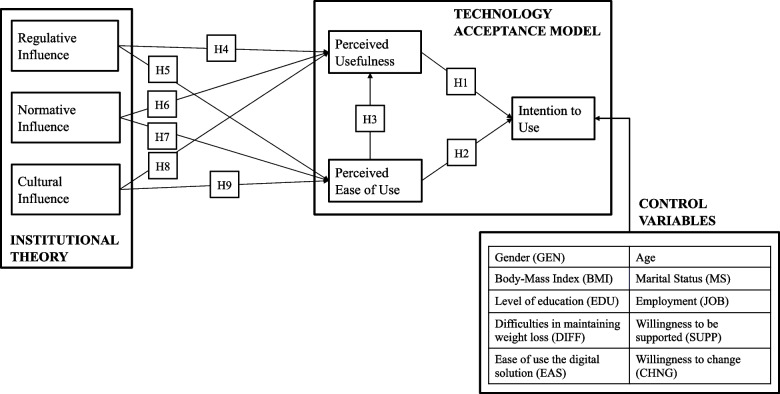
Research model

Once defined the constructs, the configuration of the model was formalized with the support of existing studies, therefore the following hypotheses were stated as follows and as shown in Fig. 1.

The basic relations of TAM were included. Specifically, the first two hypotheses test the positive influence of PU and PEU on ITU. Also, the hypothesized positive effect of the PEU on PU was included [25, 28].

Additionally, six other hypotheses were tested.

Regulative Influence is expected to impact PU by stimulating how individuals perceive the benefits (H4) [43] but also on PEU as rules can be felt as guidelines for the usage (H5). The Normative Pillar can influence PU through peer influence, as by seeing peers using technology and exploiting the benefits, one can think to have similar results (H6) [43]. Similar pressure can work on the PEU, by leveraging peer experience (H7). Cultural Influence can positively affect PEU by fostering the disposition of individuals toward the new technology and the challenges arising from it (H8). Similarly, it has been hypothesized that Cultural Influence has a positive impact on the disposition of individuals to feel that the technology is appropriate and useful (PU) (H9).

From past literature and considering the context, 10 control variables have been included in the model to see if the Intention To Use would have been influenced. These variables are gender [46, 47], age [48], marital status, Body-Mass Index (BMI), level of education [49], employment [50], difficulties in maintaining weight loss [51], ease of use of the digital solution [46], and willingness to change and to be supported [52].

### Data collection

The proposed research model questionnaire is aimed at exploring the DTx acceptance among obese patients thanks to the collaboration with Istituto Auxologico Italiano, an Italian hospital structure that is a national excellence center for obese patients’ treatment. The questionnaire has been developed and delivered to actual and former obese patients of the hospital, who had previously given their consent for being contacted for further research purposes. Furthermore, data collection has been performed in compliance with GDPR regulations, as respondents have been informed and ensured anonymity The questionnaire was delivered in two ways, an additional file shows this more in detail [see Supplementary Information.pdf]. A first paper-based version was distributed to individuals who were receiving inpatient treatments, collecting 164 high-quality responses. Health professionals supported patients in answering the questionnaire, hence, there have been no discarded or unfinished surveys. The second online version, instead, was delivered digitally by email through Qualtrics to a 3.7 k mail addresses database of patients and former patients of Istituto Auxologico. 305 patients (response rate of about 8.2%) engaged in answering, among which 167 provided complete and high-quality answers (dropout rate of 45%). By merging the results from the paper-based and the online versions a total of 331 responses have been analyzed. Given the nature of obesity as a disease, namely a chronic disease, that requires continuous and potentially life-lasting care and follow-ups, all the patients included in the sample experienced or are experiencing the institutional role of the hospital and its members. The institutional pressure exerted by an institution (i.e., the Istituto Auxologico) over the patients could be successfully observed.

The questionnaire has been divided into two parts. The first one has been dedicated to gathering general information on respondents, such as personal and demographic data, but also investigated health status, the social and familiar context, their satisfaction with past care and follow-ups, and their usage of technology. The second one measured the constructs present in the research model through items retrieved from the literature. As for institutional constructs, past research shows few examples of empirical measures for institutional constructs, which are mostly investigated through the qualitative methodology. For this reason, non-institutional items were adapted to the context. All the items have been measured through a 5-points Likert scale.

### Data analysis

**Fig. 2:**
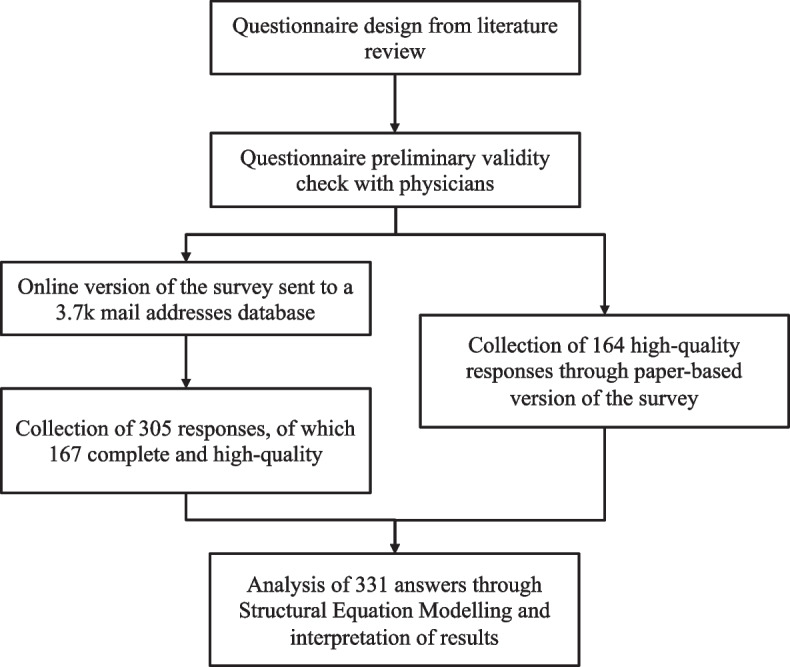
Flow chart of the methodology adopted

Once collected, the data have been analyzed. Firstly, a descriptive analysis has been performed on the questions about demographics and personal information. Secondly, the model has been tested through the software STATA 17. An overview of the flow chart describing the methodology adopted for this study is shown in Fig. 2. Specifically, a first Kaiser-Meyer-Olkin (KMO) test was applied to verify the sample adequacy for the factor analysis. Subsequently, for the first evaluation of the items measuring contracts, an Exploratory Factor Analysis (EFA) has been carried out through the Principal Component Methodology, together with Cronbach’s alpha to test the internal consistency reliability. The testing of the model went through Structural Equation Modeling (SEM), which has been proven to be an effective tool of analysis in health system research [53]. The validity and consistency of the method to measure the constructs have been assessed through the Confirmatory Factor Analysis (CFA). The convergence validity has been assessed by two indicators: composite reliability and average variance extracted.

Lastly, the goodness of fit (GOF) was proved through four indicators, both absolute like the square error of approximation (RMSEA) and the standardized root mean residual (SRMR), and incremental like comparative fit index (CFI) and the Tucker-Lewis index (TLI).

## Results

Among the 331 respondents, 71% were mainly female, around 60% of respondents are aged between 51 and 70 years old, and most of the respondents are in a situation of moderate or severe obesity (BMI among 31–50). The school level and employment of respondents are in line with the general situation in Italy. Their digital proficiency can be positively evaluated as around 45% of the respondents can easily use a smartphone and a similar proportion use digital solutions to manage their health, representing a strong possibility to use this new tool of a DTx.

The quantitative analysis started with the KMO test, with a value of 0.9332, thus showing that the factor analysis was worth it. Both EFA and CFA confirmed the validity of the relation between items and latent variables, as shown in Table 2.

**Table 2:** Constructs, measurament items and relevant measurement properties of the proposed model

Construct	Item	Measurament Item	Factor Loading	CR	AVE
Intention To Use (ITU)	ITU1	I will want to use this medical APP	0.942	0.912	0.777
	ITU2	I plan to use this medical APP in the future	0.939		
	ITU3	I would like to use this medical APP to keep fit	0.750		
Perceived Usefulness (PU)	PU1	Using this medical APP will improve my lifestyle and health	0.810	0.926	0.806
	PU2	The use of this medical APP will allow me to manage my care pathway more effectively	0.941		
	PU3	The use of this medical APP will help me to manage my health	0.938		
Perceived Ease of Use (PEU)	PEU1	Using this medical APP will NOT require much effort from me	0.822	0.886	0.722
	PEU2	Using this medical APP will be intuitive and easy for me	0.847		
	PEU3	When I use this medical APP on my mobile phone I will easily be able to do what I need to do	0.878		
Regulative Pillar (RP)	RP1	I always agree with what the doctors who treat me tell me, including the use of this medical APP	0.862	0.936	0.774
	RP2	I always agree with the priorities given to me by the doctors treating me, including on the use of this medical APP	0.894		
	RP3	I always agree with the therapies prescribed by the doctors treating me, including when using this medical APP	0.885		
Normative Pillar (NP)	NP1	People I rate most highly think I should use this medical APP to improve my care pathway	0.825	0.886	0.721
	NP2	People I estimate most would use a medical APP to improve their care pathway if prescribed by their doctor	0.858		
	NP3	People I value most think that medical apps can help improve their care pathway if certified and validated	0.865		
Cultural Pillar (CP)	CP1	In my circle of family/friends/colleagues there is full confidence in technological innovation (like, for example, this medical APP)	0.806	0.861	0.674
	CP2	There is NO fear in my circle of family/friends/colleagues to try new things (like, for example, this medical APP)	0.813		
	CP3	In my circle of family/friends/colleagues there is full openness to digital solutions (like, for example, this medical APP)	0.843		

Subsequently, the SEM validated the model applied.

Specifically, the relations between the PU and ITU, and between PEU and PU have been confirmed, while the one between PEU and ITU has been found to be not significant. NP had a significant impact on PU, but not on PEU, while RP positively affected both PU and PEU. No control variables had a significant relation with the Intention to Use. Instead, CP positively influenced PEU but was not significant on PU. All the hypotheses coefficients and the goodness of fit indexes were shown to be inside the acceptability threshold, as presented in Tables 3 and 4.

**Table 3:** Path analysis and hypotheses testing results of model constructs

Hypothesis	Path	β Coef.	td. Err.	*P*-Value
H1	PU ➔ ITU	0.830	0.057	0.000***
H2	PEU ➔ ITU	0.057	0.070	0.414
H3	PEU ➔ PU	0.491	0.058	0.000***
H4	RP ➔ PU	0.297	0.062	0.000***
H5	RP ➔ PEU	0.527	0.061	0.000***
H6	NP ➔ PU	0.145	0.066	0.028*
H7	NP ➔ PEU	0.120	0.083	0.148
H8	CP ➔ PU	0.007	0.065	0.910
H9	CP ➔ PEU	0.179	0.079	0.025*

**Table 4:** The goodness of fit indexes

Indicator	Threshold	Value
RMSEA	< 0.08	0.046
SRMR	< 0.08	0.044
CFI	> 0.9	0.961
TLI	> 0.9	0.956

## Discussion

The main theoretical contribution comes from the fact that to the best of the authors’ knowledge, the application of theoretical models to study the acceptance of DTx is not recurrent, also considering the innovativeness of the product. Additionally, the novel combination of two different frameworks, namely TAM and Institutional Theory, provides an original contribution. It has been proven that the institutional factors influence TAM constructs. The regulatory factor, indeed, contributes to the technology’s PU on the one hand, while simultaneously making it appear easier to use on the other hand. Peer influence represents a great source of confidence for the patient when dealing with a new treatment fostering the PU. In the scenario, where DTx becomes a “habit” or “ritual” through the positive cultural change, it could become simpler to utilize and approach (PEU). Additionally, the theoretical frameworks can be generalized. Indeed, the application of this model can be certainly studied for others innovative digital solutions in healthcare to understand their acceptance from users. For instance, a different DTx addressing diseases others from obesity could be interestingly studied by relying on the proposed model.

The interpretation of the results made it also possible to deduce some insightful managerial considerations, which were deeply discussed with both managers and physicians from the institute. The fact that the DTx are easy to use does not directly affect Intention To Use. The PEU, on the other hand, might be viewed as an added benefit that contributes to a higher PU. Given the potentially high benefits that could derive from effective communication, Auxologico Hospital would significantly benefit from a clear disclosure towards patients. Additionally, training or external support may be recommended to break down the barrier of unfamiliarity with digital solutions.

The regulatory factor, in particular, has a positive impact on both PU and PEU. As a result, the institution can encourage the patient to use the DTx both by leveraging on the Perceived Usefulness and by creating rules which work as guidelines. Indeed, the institution should establish a set of laws and regulations to safeguard patients while letting them feel guided during the application.

In addition, since peer influence impacts PU, one approach could be to form a community among Auxologico patients who are planning to adopt or have adopted DTx. The consequences are favorable since, on the one hand, collected feedback serves the patients to compare themselves with peers and, on the other hand, it is also beneficial to the hospital in terms of continuous improvement.

Finally, the Cultural Pillar embedded in the organization has a good impact on PEU. This latter aspect, in turn, opens reflection on interesting and necessary policy implications for properly managing Digital Therapeutics in an institutional setting, such as Istituto Auxologico Italiano. More specifically, the hospital could consider modifying the current Integrated Care Pathaways in order to include DTx as further therapeutic approaches. In assessing the most appropriate treatment, on a case-by-case basis, physicians might include the prescription for DTx for the care path of eligible patients. Therefore, the key point is to make DTx “ordinary” and “familiar” for the patient so that they can be seen as simple as possible. Digital Therapeutics should not be viewed as a niche or experimental treatment for a selected few. Instead, DTx must be open to everybody, adaptable to each condition, and expandable over time, so that the patient perceives them as basic and easy to use.

### Limitations of the study and further research

The findings revealed some weaknesses in the research. Firstly, the main constraint is due to the features of the sample examined. Indeed, 341 answers from a single healthcare facility were collected, and further research to widen the sample could be suggested by involving a wider number of hospitals (institutions) and their patients. Enlarging the sample could also allow for improving the sample characteristics. In Italy, indeed, obesity is more common among males than women, while in the current sample, women represent the majority (about 70% of the sample).

Future studies can be conducted based on the gaps highlighted by this work, with the goal of ongoing development. Firstly, the collaboration with Auxologico revealed the need to collect opinions also from physicians. Doctors, indeed, play a crucial role in the process related to the diffusion of a Digital Therapeutic, as they are involved in the prescription of DTx. Obtaining evidence of the factors related to physicians’ perception of DTx would allow for obtaining a more comprehensive perspective about the acceptance of such technologies. The current research provides first insights in this direction by looking at the role of (health) institutions, where physicians are among the main representative. Therefore, an additional step could be to administer a questionnaire dedicated to the healthcare professionals and repeat the analysis assuming this different, yet fundamental, perspective.

Given the distribution of the sample centered on mildly obese patients, and the peculiar care path dedicated to bariatric patients who instead are affected by severe obesity, a future study could differentiate between these two subgroups, focusing mainly on mild obesity which can be more effectively treated through DTx.

Additionally, thanks to the above-mentioned adaptability of the theoretical model, additional research directions could investigate the acceptability of DTx in patients affected by diseases other than obesity. Moreover, other researchers could test the applicability of the model with respect to other digital innovations in healthcare, whose acceptability and diffusion can be interestingly predicted through interaction with Institutional actors.

## Conclusions

Overall, the findings of the present study show that the proposed model can be employed to predict obese patients’ acceptance of digital technology, such as Digital Therapeutics, for the treatment of obesity via Cognitive Behavioral Therapy. The original combination of well-established, yet different, theories allows targeting both the rational and institutional factors affecting individuals’ Intention To Use DTx, which the adopted methodological approach has validated. Besides the theoretical achievements, this study also sheds light on possible practical implications, being an insightful starting point for all those professionals dealing with the timely topic addressed, namely obesity treatment.

## Supplementary Information

**Additional file 1.** Questionnaire. Supplementary material is provided in a separate file (file name: Supplementary Information.pdf). The file contains the questionnaire submitted to obese patients. More in detail, the questionnaire is made by two sections: part A (demographics and personal information) and part B (Model Measurement). Part B has been measured through Likert Scale from 1: strongly disagree to 5: strongly agree.

## Data Availability

The datasets generated and analysed during the current study are not publicly available. Although we have removed identifying information, we cannot risk identification by making the data available for public inspection, as we guaranteed anonymity to respondents. Datasets could be available from the corresponding author on reasonable request.

## Abbreviations

DTx: Digital Therapeutics
TAM: Technology Acceptance Model
ITU: Intention to Use
PU: Perceived Usefulness
POU: Perceived Ease of Use
RP: Regulative Pillar
NP: Normative Pillar
CP: Cultural Pillar
GEN: Gender
BMI: Body-Mass Index
MS: Marital status
EDU: Level of education
JOB: Employment
DIFF: Difficulties in maintaining weight loss
SUPP: Willingness to be supported
EAS: Ease of use of the digital solution
CHNG: Willingness to change
