# Supplementary material for: What drives patients’ acceptance of Digital Therapeutics? Establishing a new framework to measure the interplay between rational and institutional factors
Source: BMC Health Serv Res. 2023 Feb 10;23:145. doi: 10.1186/s12913-023-09090-7 (PMC9921481; doi:10.1186/s12913-023-09090-7)
Supplement: Supplementary file 1 — Additional file 1. Questionnaire. Supplementary material is provided in a separate file (file name: Supplementary Information.pdf). The file contains the questionnaire submitted to obese patients. More in detail, the questionnaire is made by two sections: part A (demographics and personal information) and part B (Model Measurement). Part B has been measured through Likert Scale from 1: strongly disagree to 5: strongly agree. [file 12913_2023_9090_MOESM1_ESM.pdf]

Supplementary Information

Questionnaire

The questionnaire submitted to obese patients is reported here.

Part A: Demographics and personal information

#	Question	Answers
**1**	Gender	Male
		Female
**2**	Age range	< 18
		18-30
		31-40
		41-50
		51-60
		61-70
		>70
**3**	Weight (kg)	………………..
**4**	Height (cm)	………………..
**5**	What is your highest level of education	Elementary
		Lower middle school
		High school
		Bachelor's degree
		Doctoral or postgraduate degree
**6**	Region of residence	………………..
**7**	What is your occupation?	Student
		Worker
		Unemployed
		Housemaker
		Retired
**8**	What romantic relationship are you in?	Single
		I have a stable partner
		Other
**9**	How much do you agree with the following statement: "I consider myself a person who likes to be in company"?	Strongly disagree
		Disagree
		Neutral
		Agree
		Strongly agree
**10**	With whom do you consume the main meals of the day?	Alone
		With family
		With colleagues/friends
**11**	Does your household support/motivate you in maintaining/improving your health status?	Yes
		No
**12**	Does your household NEGATIVELY influence your eating behavior?	Yes
		No

**13**	Is/would your household be involved in your care journey?	Yes
		No
**14**	How much do you agree with the following statement: "I am the main architect of my own health"?	Strongly disagree
		Disagree
		Neutral
		Agree
		Strongly agree
**15**	How much do you agree with the following statement: "I use Apps on my cell phone (smartphone) with great ease"?	Strongly disagree
		Disagree
		Neutral
		Agree
		Strongly agree
		I don't have a cell phone with access to the Internet and Apps (smartphone)
**16**	Are you affected by one or more of these conditions? (You can select more than one answer)	Cardio or cerebrovascular disease
		Hypertension
		Diabetes
		Liver disease
		Osteoarthritis
		Osteoporosis
		Kidney disease
**17**	Are you taking or have you taken drug therapy for obesity?	Yes
		No
**18**	Have you performed any of the following health care services in the past year? (You can select more than one answer)	Ambulatorio (Obesity Center)
		MAC / Day Hospital
		Hospitalization (e.g., Piancavallo)
		Bariatric Surgery
**19**	How much do you agree with the following statement: “My health status improved while being taken care of by Auxologico”?	Strongly disagree
		Disagree
		Neutral
		Agree
		Strongly agree
**20**	How much do you agree with the following statement: “I am satisfied with the results obtained”?	Strongly disagree
		Disagree
		Neutral
		Agree
		Strongly agree
**21**	Once you return home, do you have difficulty managing the maintenance/improvement of results over time?	I have no difficulty (I manage well on my own without external support)
		Yes, I have difficulty in the area of weight maintenance/reduction
		Yes, I have difficulty in the area of eating habits/behavior
		Yes, I have difficulty in the area of motivation to change
		Yes, I have difficulties in other aspects
**22**	Would you like to be able to be followed by health care	I don't care
		Yes, for 1 month

	professionals remotely from home, even through technological-digital tools?	Yes, for 3 months
		Yes, for 6 months
		Yes, for 12 months
		Yes, for a lifetime
**23**	How many hours a day do you use your cell phone (smartphone)?	Less than 1 hour
		1-2 hours
		3-5 hours
		5-10 hours
		More than 10 hours
**24**	Do you use digital solutions (e.g., APPs, wearable devices such as smartwatches, smart bracelets) to manage your health (e.g., to track a diet or exercise)?	Never
		Rarely
		Sometimes
		Often
		Always
**25**	Do you know an APP called "Noom?" (Please, read all the possible answers carefully)	Never heard of it
		Heard about it from some of my family/friends/colleagues, but never downloaded it
		Heard about it from some doctors, but never downloaded it
		I heard about it on the web/social media, but never downloaded it
		I heard about it in some magazines and downloaded it to try it out
		I heard about it from some of my family/friends/colleagues and downloaded it to try it out
		I heard about it from some doctors and downloaded it to try it out
		I heard about it on the web/social media and downloaded it to try it out
		I heard about it in some magazines, but never downloaded it
**26**	Do you know of APPs for "intermittent fasting" ("fasting" in English) such as Fasting; DoFasting; Zero; Vora; 21 Day Hero? (Please, read all possible answers carefully)	Never heard of it
		Heard about it from some of my family/friends/colleagues, but never downloaded it
		Heard about it from some doctors, but never downloaded it
		I heard about it on the web/social media, but never downloaded it
		I heard about it in some magazines and downloaded it to try it out
		I heard about it from some of my family/friends/colleagues and downloaded it to try it out
		I heard about it from some doctors and downloaded it to try it out
		I heard about it on the web/social media and downloaded it to try it out
		I heard about it in some magazines, but never downloaded it

Part B: Model Measurement through Likert Scale (from 1: strongly disagree to 5: strongly agree)

#	Question	1	2	3	4	5
**1**	I will want to use this medical APP					
**2**	I plan to use this medical APP in the future					
**3**	I will want to take advantage of this medical APP to keep myself fit					
**4**	Using this medical APP will improve my lifestyle and health					
**5**	Using this medical APP will allow me to more effectively manage my care pathway					
**6**	Using this medical APP will be helpful in managing my health					
**7**	Using this medical APP will NOT require a great deal of effort on my part					
**8**	Using this medical APP will be intuitive and easy for me					
**9**	When I use this medical APP on my cell phone, I will easily be able to do what I need to do					
**10**	I always agree with what the doctors who are treating me tell me, including about using this medical APP					
**11**	I always agree with the priorities given to me by the doctors in my care, including on the use of this medical APP					
**12**	I always agree with the therapies that the doctors who treat me prescribe, including on the use of this medical APP					
**13**	People I value most think I should use this medical APP to improve my course of treatment					
**14**	People I value most would use a medical APP to improve their care pathway if prescribed by their doctor					
**15**	People I value most think that medical APPs can help improve the care pathway if certified and validated					
**16**	In my circle of family/friends/colleagues, there is full confidence in technological innovation (such as, for example, this medical APP)					
**17**	In my circle of family/friends/colleagues there is NO fear of trying new things (like, for example, this medical APP)					
**18**	In my circle of family/friends/colleagues there is full openness to digital solutions (like, for example, this medical APP)					
